# The Role of Cell-Penetrating Peptide and Transferrin on Enhanced Delivery of Drug to Brain

**DOI:** 10.3390/ijms17060806

**Published:** 2016-05-25

**Authors:** Gitanjali Sharma, Sushant Lakkadwala, Amit Modgil, Jagdish Singh

**Affiliations:** 1 Department of Pharmaceutical Sciences, College of Health Professions, North Dakota State University, Fargo, ND 58105, USA; gitanjali.sharma@ndsu.edu (G.S.); sushant.lakkadwala@ndsu.edu (S.L.); 2 Department of Neuroscience, Tufts University School of Medicine, 136 Harrison avenue, Boston, MA 02111, USA; amit.modgil@tufts.edu

**Keywords:** cell-penetrating peptide (CPPs), transferrin, blood brain barrier (BBB), liposomes, functionalization

## Abstract

The challenge of effectively delivering therapeutic agents to brain has led to an entire field of active research devoted to overcome the blood brain barrier (BBB) and efficiently deliver drugs to brain. This review focusses on exploring the facets of a novel platform designed for the delivery of drugs to brain. The platform was constructed based on the hypothesis that a combination of receptor-targeting agent, like transferrin protein, and a cell-penetrating peptide (CPP) will enhance the delivery of associated therapeutic cargo across the BBB. The combination of these two agents in a delivery vehicle has shown significantly improved (*p* < 0.05) translocation of small molecules and genes into brain as compared to the vehicle with only receptor-targeting agents. The comprehensive details of the uptake mechanisms and properties of various CPPs are illustrated here. The application of this technology, in conjunction with nanotechnology, can potentially open new horizons for the treatment of central nervous system disorders.

## 1. Introduction

Over recent years, there has been a considerable progress in the field of neuroscience leading to an improved understanding of disorders of the central nervous system (CNS). In contrast, the development of successful strategies for treating these disorders is limited due to the protective function of the blood brain barrier (BBB). The concept of the BBB originated in late 19th and early 20th centuries, when Paul Ehrlich and his colleagues discovered that some dyes could stain the brain cells after direct injection into brain, but were not able to penetrate brain after peripheral administration [1]. A parallel study revealed that bile salts induced seizures after direct injection into brain, but did not show any related symptoms after peripheral administration [2,3]. Since then, there have been comprehensive efforts to provide a scientific definition of BBB and to elucidate the mechanisms of the transport of different molecules across this barrier [4,5,6,7]. More restrictively, BBB is defined as the microvasculature of brain that is composed of endothelial cells having tight intracellular junctions and the absence of any fenestrae [4].

However, the vascular BBB does not explain all facets of conceptual BBB. The choroid plexus with cerebrospinal fluid, referred to as the cerebrospinal fluid barrier (CSFB), is another important gateway to reach brain parenchyma [8,9]. The entry of any molecule into brain, after parenteral administration, is largely controlled by BBB and CSFB [7]. However, the CSFB faces a ventricle that flushes the drug, injected in the back of the ventricle, back into the blood [10,11]. Moreover, there are about 100 billion capillaries with a surface area of 20 m^2^ that contribute to the formation of BBB [12]. Therefore, BBB is universally considered the most important barrier in restricting the transport of molecules into brain [13]. There has been an increasing incidence of the use of cell-penetrating peptides (CPPs) in the transport of therapeutic molecules into brain. CPPs are short cationic peptides (less than 30 amino acids) that have the ability to transport extracellular molecular cargo into the cells. These peptides are capable of entering the cells without producing cytolytic effects. In addition, they effectively bypass P-glycoprotein (P-gp) in the BBB and are therefore used as vectors for the delivery of drugs that are substrates for P-gp [14]. The CPPs are primarily considered to be transported via energy-dependent pathways, but the exact mechanism is not yet fully understood. While for some CPPs, endocytosis is the exclusive mechanism of uptake, for others, it is an alternative mechanism [15]. The use of CPPs is based on the fact that these peptides can be linked to the therapeutically-active molecules and can be transported across the cell membrane. This linkage can either be covalent or non-covalent. Various cargo molecules and delivery agents have been delivered into the cells using CPPs, e.g., proteins, small drug molecules, nucleic acids, liposomes and nanoparticles. The major challenge of using CPPs as transporters of therapeutic molecules is their non-targeting disposition. The combination of these CPPs with receptor- or protein-targeting moieties can actively deliver the molecules of interest into desired cells in sufficient concentrations [16,17,18].

## 2. Role of the Blood Brain Barrier in Reducing the Penetration of Molecules into Brain

**Figure 1:**
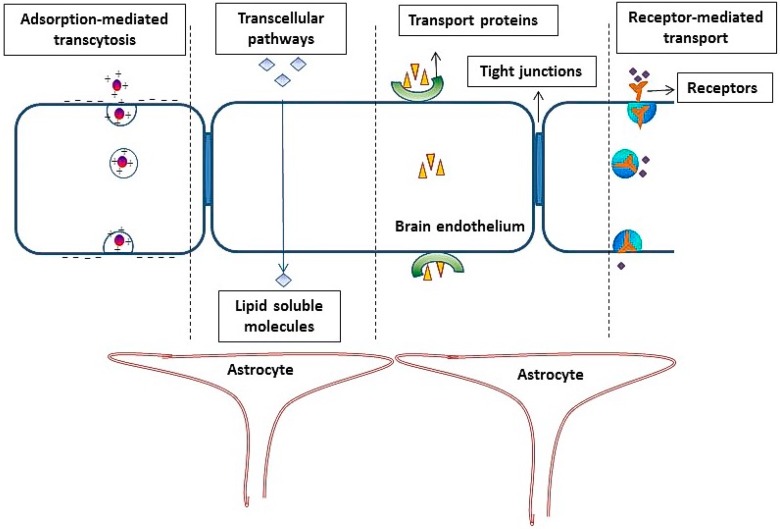
Schematic representation of the blood brain barrier and various transport processes across the brain endothelial layer.

The high impermeability and selectivity of the blood brain barrier prevents the transport of many drugs and other therapeutic molecules into brain [19,20]. The delivery of therapeutic agents across BBB has engendered substantial interest over the past few decades [9,21,22]. BBB possesses unique biological characteristics that contribute to restricting the movement of molecules to brain (Figure 1):

(1) Brain endothelial cells lack fenestrations, have very few pinocytotic vesicles and a larger number of mitochondria [23,24,25].
(2) The occurrence of an intricate complex of transmembrane proteins (e.g., occludins, claudins), forming intimate intracellular connections, called tight junctions (TJ).
(3) The expression of different transport proteins on brain endothelial cells, like *p*-glycoproteins (efflux transporters) and multidrug resistance proteins (MRPs) [10,26].
(4) The synergistic influence of astrocytes, pericytes, astrocytic perivascular end feet, macrophages and neurons on BBB functions [27,28,29].

The immune barrier of brain is formed by tightly-packed endothelial cells, perivascular macrophages and mast cells and is further reinforced by the macroglial cells. This barrier limits the passage of external immune cells, especially lymphocytes, across BBB [30,31,32,33,34].

This unique environment of CNS presents a formidable barrier to the delivery of a wide number of therapeutic molecules to brain. With the exception of small lipophilic drug molecules having a molecular mass of less than 400–600 Da, most of the drugs in circulation do not penetrate the BBB [34,35]. More than 98% percent of the drugs are halted mid-development due to poor brain permeability [36]. Furthermore, recent statistics from the National Cancer Institute show about 22,910 new cases of brain tumor leading to 13,700 deaths, each year. In addition, neuro-degenerative diseases like Alzheimer’s disease have become the most common cause of dementia among the elderly and have been reported to affect about 5% of Americans over the age of 65 and 20% over the age of 80 years [37]. These factors have triggered extensive efforts, by scientists across the globe, in developing safe and efficient vectors for the delivery of therapeutics to brain. Viruses are equipped with different molecular mechanisms to overcome these hurdles and can, therefore, serve as efficient delivery vectors [38]. Yet, the potential application of viruses as delivery agents and their further investigation in clinical research is impeded by the associated immune response and cytotoxicity, thereby accentuating the need for the synthesis of safe and efficient non-viral delivery vectors. Multidisciplinary approaches involving biology, nanotechnology and biophysics need to be considered to accomplish the goal of improving the delivery of therapeutic drugs and genes across BBB [7].

Certain inflammatory conditions of brain, for example Alzheimer’s disease, multiple sclerosis, tumors and stroke, have been known to increase the permeability of BBB. Various inflammatory mediators associated with these CNS disorders can modulate the tight junctions and enhance the passage of molecules across brain endothelium. Apart from the modulation of tight junctions on the endothelium, other closely-associated cells, such as pericytes, astrocytes, mast cells, glial cells and neurons, are also reported to be involved in inflammatory reactions [39]. Many inflammatory agents, like bradykinin, serotonin and histamine, increase leakage across blood brain barrier by increasing both endothelial permeability and vessel diameter, thereby causing cerebral edema.

Lopez-Ramirez *et al.* [40] recently reported that a molecule called “microRNA-155” is responsible for cleaving epithelial and endothelial cells. This cleavage can create microscopic gaps in the endothelium, leading to increased permeability of BBB. This discovery has opened a completely new platform for developing therapies that can help penetrate the BBB and deliver potential agents for the treatment of CNS disorders [40].

## 3. Cell-Penetrating Peptides

Cell-penetrating peptides (CPPs) are short cationic or amphipathic peptides that have the ability to transport the associated molecular cargo (e.g., peptides, proteins, oligonucleotides, liposomes, nanoparticles, bacteriophages, *etc.*) inside the cells [16]. Biological evolution has conferred certain proteins with an ability to penetrate the cell membrane due to the presence of specific peptide sequences, called protein transduction domains [41]. The peptide sequences constituting these domains carry basic amino acids and possess cell-penetrating properties; thus, these peptides are referred to as cell-penetrating peptides (CPPs). Over the past decade, there has been a vibrant increase in the application of these CPPs for the delivery of cargo molecules inside the cells [42]. These peptide sequences have been utilized for the delivery of various molecules, like proteins, nucleic acids, liposomes and nanoparticles, across the cell membrane [43,44,45,46,47]. The profound interest evoked by the CPPs among scientists is not only attributed to their ability of crossing the cell membrane via receptor and energy-independent processes, but also their capacity to efficiently internalize the associated biomolecules without compromising their biocompatibility [48]. Various CPPs, such as poly-l-arginine (PR), transactivator of transcription peptide (TAT) [49] and penetratin, have been conjugated to the delivery vectors to improve the delivery of therapeutic molecules [50,51,52].

### 3.1. Poly-l-Arginine

Polyarginine is a synthetic cationic peptide consisting of eight or more arginine residues and has been used to facilitate the intracellular translocation of a wide variety of molecular cargo [16,17]. This cell-penetrating peptide has been used for the delivery of cargo, such as liposomes, nucleic acids, nanoparticles, *etc.*, into the cells. Kibria *et al.* [50] showed that dual modification of liposomes with polyarginine and cyclic RGD (Arg-Gly-Asp) peptide significantly increased the transfection efficiency of liposomes in integrin α(v)β(3)-expressing cells. Later, Opanasopit *et al.* [53] demonstrated considerable improvement in the transfection efficiency of liposomes after coating with poly-l-arginine. A previous report provided a deeper insight into the interaction of cationic peptides with the phospholipid bilayer during the surface adsorption of positively-charged amino acids onto the liposomal surface [54]. The results showed that the adsorption of cationic amino acids, like arginine, was not only driven by electrostatic interactions, but also by polarization forces and caused surface rearrangements in the phospholipid membrane. Zhang *et al.* [55] showed that siRNA-containing octaarginine-modified liposomes efficiently inhibited the targeted gene and significantly reduced the tumor cell proliferation.

### 3.2. HIV-1 Trans-Activator of Transcription Peptide

TAT is a protein encoded by the TAT gene of HIV-1. TAT was discovered with the emergence of various CPPs of natural (AntP/penetratin) and synthetic (mastoparan/transportan) origin that have been alternatively termed as protein transduction domains (PTDs) [56,57]. Over recent years, TAT peptide has gained significant attention in the field of nucleic acids and drug delivery. A previous study compared the transfection efficiencies of the SLN gene delivery vector and polyethylenimine (PEI), *in vitro* and *in vivo*. The presence of TAT significantly enhanced the gene expression of SLNs in different cell lines as compared to the PEI nanoparticles [58]. Another group of scientists reported efficient gene delivery using TAT peptide-functionalized polymeric nanoparticle complexes into undifferentiated and differentiated SH-SY5Y cells [59]. TAT peptide-modified liposomes showed considerable improvement in the delivery of plasmid-encoding green fluorescent protein (pGFP) to human brain tumor U-87 cells *in vitro* and in an intracranial tumor mice model [60]. TAT-modified liposomes synthesized with small quantities of the cationic lipid, dioleoyl trimethylammonium propane (DOTAP) showed substantially higher gene expression levels in mouse fibroblast NIH3T3 and cardiac myocyte H9C2 cells and lower cytotoxic potential as compared to the commercially available transfecting reagent Lipofectin^®^ [45,61]. Despite the large area of application of the TAT peptide, the exact mechanism of its cellular internalization still appears controversial. Variable results illustrating different mechanisms of uptake can result from variation in different experimental factors, like the wide range of the sequences of TAT peptide used, variable cell lines and different protocols for the investigation of the mechanism of entry, which can influence the mechanism of internalization of TAT peptide.

### 3.3. Penetratin

Penetratin is a 16-amino acid basic cationic CPP, derived from the antennapedia homeodomain, which is capable of inducing the cell uptake of a large variety of molecular cargo [61]. The peptide is translocated across the cell membranes by the third α-helix of the homeodomain of antennapedia, known as penetratin. Previous biophysical studies have shown that even though the entry of this peptide requires initial binding to the cell membrane, binding and translocation are differentially affected by the amphiphilic nature and net charge of the peptide. Furthermore, the internalization of penetratin is affected by the lipid composition of the plasma membrane [62,63]. A group of researchers showed that the presence of negatively-charged lipids in the membrane promote the transfer of penetratin from a hydrophilic to a hydrophobic environment likely via charge neutralization. They showed that the transfer of penetratin can also occur in the absence of the negatively-charged lipid by adding DNA oligonucleotides, by the same mechanism. Their findings further confirmed that charge neutralization and phase transfer represented only the initial step of internalization, while further uptake required the presence of tryptophan at position 6 of the peptide [64]. A previous study showed enhanced accumulation of penetratin-functionalized PEG-(poly lactic acid) (PLA) nanoparticles in rat brain and low uptake by non-specific organs, as compared to the protamine-conjugated nanoparticles [65]. Another group of researchers showed improved transfection efficiency of penetratin-conjugated polymethacrylates as compared to PEI-polymethacrylates and comparable to the gene expression of Lipofectamine^®^ [66]. Conjugation of penetratin with elastin like polypeptides showed maximum reduction in growth and proliferation of human ovarian carcinoma cells (SKOV-3) and HeLa cells [67].

### 3.4. Mastoparan

Mastoparan is a 14-residue peptide from wasp (*Vespula lewisii*) venom and belongs to a class of peptides that are more amphipathic [68,69]. This peptide has been used in the construction of 21-residue peptide transportan 10 (TP10), which has been widely investigated in the delivery of cargo like proteins into cells [70]. The use of this amphiphilic peptide is restricted due its cytolytic effects [71,72,73]. Previous reports have indicated the application of mastoparan peptide for mitochondrial delivery, causing increased apoptosis of tumor cells [74,75]. Yamada *et al.* reported that the peptide caused increased permeability of the mitochondrial membrane, causing the leakage of components from the mitochondrial matrix, eventually leading to apoptosis of tumor cells [76]. Another report showed that the presence of mastoparan peptide, transportan 10 (TP10), significantly increased the transfection efficiency of PEI. Furthermore, a low concentration (0.6 nM) of TP10 conjugates with DNA showed efficient gene expression in HeLa cells and murine fibroblast C3H 10T1/2 cells [77].

Despite the significant advantage of using CPPs for increasing the transport of molecular cargo across the cellular barriers, these highly-efficient carriers have had controversy due the associated toxicity at high concentrations. The delivery of small molecules, vectors and other protein and nucleic acid drugs that are associated with poor brain penetration can be efficiently transported across BBB via adsorptive-mediated transcytosis (AMT). However, the non-specific uptake of the CPP or cationic proteins can result in higher accumulations in blood vessels and peripheral organs. In addition, the toxicity and immunogenicity associated with chemical modifications of proteins can pose a challenge to the practical application of these agents in improving brain delivery. Previous studies have reported membrane toxicity and tissue inflammation using CPPs and cationic albumin nanoparticles [15]. A recent report has indicated that it is non-toxic up to a concentration of 100 µM; however, after, peptide-bound TAT demonstrated significant and chain length-dependent toxicity irrespective of the sequence of peptide [73]. Another study indicated toxicity associated with a very high dose of the TAT_46–60_ peptide [78].

## 4. Adsorptive-Mediated Transcytosis

The growing evidence indicating the success of the transport of therapeutic molecules into brain via cationic proteins and cell-penetrating peptides (CPPs) has conveyed significant importance to AMT as the route for the delivery of molecules across BBB. Table 1 illustrates commonly-used CPPs with their features. Despite the variation in length and sequence of amino acids, these peptides share some common features, like their amphipathic nature, net positive charge, theoretical hydrophobicity and helical moment, the ability to interact with lipidic membranes and to adopt a distinct secondary structure upon association with lipids [71]. Adenot *et al.* have previously reported an increase in the penetration of various chemotherapeutic agents across BBB in *in situ* and *in vitro* cell-based models, after conjugation with SynB3 CPP [79]. They reported an increase in brain delivery of doxorubicin by a factor of 30, benzylpenicillin by a factor of seven, paclitaxel by a factor of 22, dalargin by a factor of 18 and morphine 6-glucoronide by a factor of 50 without disrupting the function of BBB. Another study reported a significant increase in the uptake of dalargin after conjugation with SynB on intravenous injection into mice [80]. In addition, TAT-conjugated nanoparticles and liposomes have been used for the delivery of therapeutic agents across BBB. Qin *et al.* reported enhanced penetration of cholestrol-PEG2000-TAT into brain as compared to cholesterol-PEG2000 and conventional cholesterol liposomes [22]. Sharma *et al.* reported enhanced penetration of CPP-transferrin liposomes into brains of adult Sprague Dawley rats as compared to transferrin-conjugated and conventional plain liposomes [16,17]. A recent study demonstrated significantly (*p* < 0.001) higher accumulation of penetratin-functionalized PEG-PLA nanoparticles in rats as compared to the low molecular weight protamine nanoparticles [65].

Apart from the CPPs, cationic proteins have also been employed to increase the penetration of therapeutic agents across BBB via an adsorptive-mediated mechanism. Poduslo and Curran [81] demonstrated that polyamine modification of proteins (albumin, insulin and IgG) dramatically increased their penetration across BBB. The permeability of insulin increased by 1.7–2.0-fold; albumin increased by 54–165-fold; and IgG increased by 111–349-fold [81]. A previous study compared cationic bovine serum albumin-conjugated PEG-PLA nanoparticles (CBSA-NP) with the native bovine serum albumin-conjugated NP (BSA-NP) and unconjugated nanoparticles (NP) for brain delivery in mice [82]. The results demonstrated that the penetration of CBSA-NP increased by 2.3-fold as compared to NP. Although cationic proteins have shown considerable improvement in the delivery of molecules or delivery vectors across BBB, toxicity or immunogenicity associated with this chemical modification cannot be ruled out [37].

However, one of the difficulties for brain delivery is the poor ability of the formulation to escape from endosomes, which leads to the degradation or accumulation of drug moieties inside brain endothelial cells. This can be overcome by either the use of a pH-sensitive formulation or the use of cationic molecules [83,84]. The mechanism behind the use of the pH-sensitive formulation is the destabilization of the endosomal membrane by fusogenic peptides. The fusogenic peptides undergo conformational changes upon the change in pH, which leads to lipid merging and ultimately endosomal membrane fusion [85,86]; while in the case of cationic molecules, the binding of cationic molecules to the endosomal membrane causes thinning of the chain region and creates an internal membrane tension. This tension in the membrane is strong enough to create pores in the endosomal membrane [87]. It has been reported earlier that caveolin-1 protein assists in transcytosis across the endothelial layer [16,88].

## 5. Receptor-Mediated Transcytosis

Receptor-mediated transcytosis (RMT) overcomes the limitation of the non-specific uptake by peripheral tissues and blood vessels, thus reducing the side effects associated with AMT. Upregulation of certain receptor types in a diseased condition further enhances the opportunity for active targeting of therapeutic molecules to specific sites and tissues, e.g., transferrin receptors are overexpressed on brain endothelium, and the receptor expression is significantly upregulated in tumor conditions [46,91]. Cargo molecules, like proteins, peptides and delivery vectors, can be linked with an active targeting ligand and transported across BBB via RMT. Therefore, this approach is also called as the Trojan horse approach [12,92]. Increased understanding of BBB biology and genomics has led to the discovery of a large number of transporters and receptors that can be used for the delivery of molecules across BBB. In general, there are three steps involved in RMT [12,92]:

(1) Endocytosis of the molecules on the luminal (blood) side after binding of the ligand to the targeted receptor.
(2) Movement of the molecules across the endothelial cytoplasm.
(3) Exocytosis of the drug/ligand-attached drug or delivery vector on the abluminal (brain) side.

The second step sometimes leads to endosomal/lysosomal degradation of the drug molecules or cargo. This fate is overcome by using pH-sensitive liposomes or cationic peptides/molecules [83,84]. Certain targeting ligands, like diphtheria toxin, have endosomal escaping ability [93]. Advantageously, the lysosomal/endosomal escaping phenomenon is not required for brain delivery and successful transport of small drug molecules, liposomes, nanoparticles and polymeric complexes to brain [93,94,95,96,97].

Transferrin receptors are the most commonly-targeted receptors for the delivery of therapeutic agents to brain. The role of transferrin receptors in BBB transport is given below.

### Transferrin Receptors

**Figure 2:**
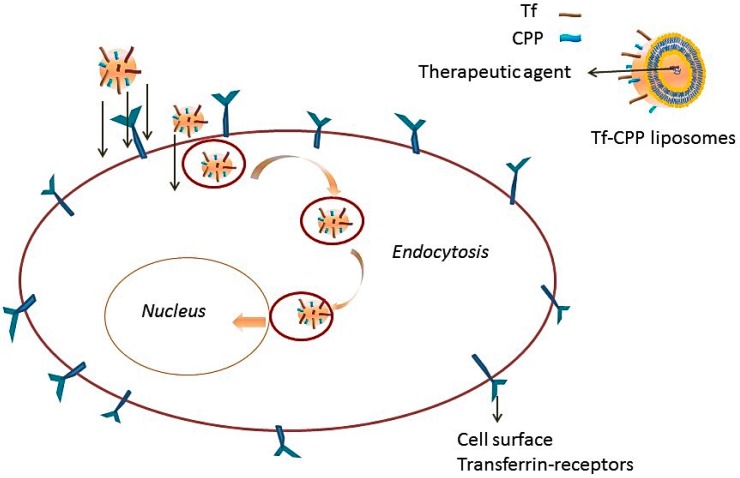
Uptake of liposomes by cells via a dual-mechanism involving receptor targeting and cell penetration.

Transferrin receptors (TfR) are the widely-studied systems for the RMT of delivery vectors and therapeutic agents across BBB. The receptor is a transmembrane glycoprotein with two subunits of 90 kDa that are linked by a disulfide bridge, and each of these subunits can bind to one molecule of transferrin [98]. In addition to the BBB, this TfR is also expressed on hepatocytes, monocytes, erythrocytes, intestinal cells, epithelial cells of choroid plexus and neurons. TfRs on BBB mediate the transport of iron bound to transferrin into the brain. Nanoparticles, liposomes and therapeutic drug molecules can be conjugated to either transferrin protein or transferrin monoclonal antibody (OX-26). The TfR-targeted monoclonal antibody binds to a different site as compared to transferrin protein and, therefore, is less likely to interfere with the endogenous transferrin in circulation. A recent study reported an improvement in the expression of luciferase gene in C6 glioma cells, primary hippocampal neurons and primary cortical neurons on transfection with transferrin-modified cationic liposomes, as compared to conventional plain liposomes. However, the transfection levels were low with the conjugation of transferrin protein. There low transfection levels with the transferrin-conjugated delivery vector were attributed to the high concentration of transferrin protein in circulation, which competes with the transferrin on the nanoparticle system [99]. Another limitation of using transferrin as a delivery system is that exogenously-supplied transferrin can lead to an overdose of iron transport into brain. In order to avoid the limitations of using transferrin as a delivery system, transferrin receptor-targeted antibodies have been used that bind to a receptor binding site differently compared to the transferrin protein. Different antibodies that have been evaluated include OX26 (anti-rat TfR monoclonal antibody), R17-217 and 8D3 (anti-mouse TfR monoclonal antibody) were all examined. Comparison of the brain uptake of R17-217 and 8D3 revealed a higher uptake of 8D3 (3.1% injected dose/gram of tissue) as compared to R17-217 (1.7% injected dose/gram of the tissue) [100]. Ulbrich *et al.* investigated the distribution and brain targeting properties of human serum albumin nanoparticles conjugated to transferrin protein or transferrin monoclonal antibodies (OX26 or R17-217) for the delivery of loperamide (does not cross BBB) [101]. The results demonstrated significant anti-nociceptive effects with loperamide-loaded human serum albumin HSA nanoparticles after covalent modification with transferrin or antibodies (OX-26 or R17-217). The study also showed enhanced transport of transferrin monoclonal antibody-modified nanoparticles across BBB as compared to the IgG2a antibody or transferrin-modified nanoparticles, thus further confirming the efficacy of monoclonal antibodies over transferrin protein for the delivery of therapeutic agents to brain [101]. A recent report showed a comparison of different targeting ligands in improving the transport of molecules to brain. Five different targeting ligands were compared for their ability to target brain, both *in vitro* and *in vivo*: transferrin, R17-217 (against TfR), COG 133 (against low density lipoprotein receptor (LDLR) and lipoprotein receptor protein (LRP)), angioprep-2 (against LRP) and cross-reacting material (CRM) 197 (against diphtheria toxin receptor (DTR)). The *in vitro* results showed that only R17-217 and CRM197 were observed to be associated with human endothelial cells, and only R17-217 showed enhanced brain uptake of liposomes in Balb/c mice at all time points after intravenous injection [102]. The authors studied the distribution of ^3^H-labelled liposomes in brain capillaries using the capillary depletion method and observed that the distribution of R17-217 liposomes was 10-times more than the untargeted liposomes. In addition, R17-217 liposomes were the only ones whose concentration was maintained in the brain over a period of 6 h and was 0.18% of the injected dose/gram of tissue after 12 h. The authors also suggested that the higher accumulation of this antibody in comparison to the other groups could be due the higher molecular weight and higher affinity of the antibody to the receptors, leading to a stronger brain targeting ability and a lower rate of elimination [102]. Although the targeting ligand has a significant contribution in improving the delivery of molecules to brain, there are other parameters, like matrix material, particle size, surface properties and the density and conformation of targeting ligand, that also play an important role in brain delivery. A recent study performed by Sharma *et al.* also showed higher accumulation of transferrin-CPP-modified liposomes in rat brain after 24 h of intravenous administration [17]. The authors proposed a dual mechanism for improved and targeted delivery of transferrin-modified liposomes (Figure 2). The conjugation of CPP with transferrin liposomes enhanced the penetration of transferrin liposomes into brain by overcoming receptor saturation *versus* the transferrin-conjugated or untargeted liposomes. Transferrin (Tf) has been evaluated to be an important target for delivery to brain. However, more studies need to be conducted in order to fully understand the function and performance of targeting ligands. The expression of TfR on brain endothelial cells was observed to decrease in brain ischemia [103]. In contrast, the expression was observed to be decreased in the hippocampus of patients with AD, as compared to normal humans [104,105]. However, there was a marked increase in the expression of transferrin receptors during brain injury and after intra-cerebral hemorrhage [106].

Several studies have been performed to show the overexpression of TfR on brain endothelial cells [107,108]. The TfR is expressed, with 10,000–100,000 molecules per cell commonly found on proliferative brain endothelial (bEnd.3) cell lines in culture [109,110]. Such a high density of TfR at BBB will facilitate the greater transport of drug encapsulated and surface-modified with Tf and CPP liposomes across the BBB. Additionally, an immunohistochemical evaluation of normal brain tissue and tumor biopsy revealed a differential staining pattern for TfRs on brain endothelial cells of normal and tumor tissue, indicating a higher level of expression in tumor-associated brain endothelial cells [111].

Various *in vitro* cell culture-based and *in situ* models have been used in the past to study the transport of molecules across the barrier layer. Despite the simplicity of the construction of the *in vitro* models, these models are limited by the lack of features, like cell-cell interactions generated in *in situ* models (e.g., induction, signaling, *etc.*). Traditionally, the transport of dyes, such as Evans Blue-albumin and fluorescein, measuring the permeability coefficient of the brain endothelium for small radiolabeled molecules, such as sucrose and mannitol, have been used [16,39]. More recently, microelectrode techniques, cable analysis and transendothelial electrical resistance (TEER) instruments have been used to determine the resistance across the endothelial barrier [16].

## 6. Nanocarriers for Delivery across BBB

These are nanoscale carriers for the delivery of therapeutic drugs or other molecules and consist of particles in the size range of 100–1000 µm [112]. These nanocarrier systems include polymeric nanoparticles and lipid-based particles, e.g., liposomes and solid lipid nanoparticles. This emerging class of delivery systems can be easily customized to transport desired therapeutic agents to specific tissues in the body. With the rapid development in polymer chemistry and nanotechnology coupled with an increased understanding of the molecular biology of brain and various receptor systems that can be used to target brain, the development of nanocarriers for delivery to brain has gained the increasing attention of scientists across the world. They can be surface modified for targeting specific receptors, can carry the therapeutic drugs and molecules in sufficient amounts and provide a controlled/targeted release of the therapeutic agent. Ideal nanocarriers should have the following properties for the delivery of drugs/therapeutic agents across BBB [113,114]:

(1) They should be biodegradable, non-toxic and biocompatible.
(2) They should preferably have a size of less than 200 nm.
(3) They should not aggregate/dissociate in blood and should be stable in circulation.
(4) They should be non-immunogenic.
(5) They should have a targeting moiety coupled for delivery across BBB via receptor/adsorptive transcytosis or monocytes and macrophages.
(6) The drug (small molecules, peptides, proteins, nucleic acids) carried should be stable, and the drug release should be tunable.

A large variety of nanocarriers have been developed so far; however, only polymeric nanoparticles and amphiphilic lipids forming liposomes have been extensively exploited for the delivery of therapeutic agents to brain [115]. Table 2 shows various nanocarriers for drug and gene delivery to brain. Several polymeric and liposomal delivery systems for the treatment of brain disorders have reached clinical trials. The University of Regensburg in collaboration with Essex Pharma (Schering-Plough) has successfully completed phase 2 of clinical trials for PEGylated liposomal doxorubicin and prolonged temozolomide in combination with radiotherapy in the treatment of glioblastoma [7,116]. Another PEGylated doxorubicin formulation surface modified with glutathione is currently in phase I/II clinical trials in the Antoni van Leeuwenhoek hospital, the Netherlands. Non-amphiphilic colloidal drug carriers, like dendrimers and micro-emulsions, are still at relatively early stages of development.

### 6.1. Liposomes

Liposomes are lipid vesicles that have an inner aqueous core surrounded by a phospholipid bilayer. The pulsating development of targeted nanoparticulate systems has allowed efficient delivery of therapeutic agents to brain [51]. Various nano-constructs, like liposomes, dendrimers, lipid-polymeric nanoparticle systems and nanocapsules, have been evaluated in the recent past for the delivery of desired cargo to the target site [135,136,137,138,139]. The versatility of liposomes, their ability to efficiently protect the encapsulated therapeutic agent in circulation and the simplicity of surface engineering provide a substantial advantage to the liposomal delivery vectors over other nanoparticle systems [140,141]. These liposomes can be conjugated to proteins for targeting specific receptors. Furthermore, sterically-stabilized liposomes, surface modified with polyethylene glycol (PEG), show a reduction in clearance by the reticuloendothelial system and immunogenic response of the targeting proteins [142,143]. Low elimination by the liver and spleen increases the circulation time of liposomes and improves the bioavailability of encapsulated molecules for therapeutic action [144,145].

#### Functionalization with Ligands for Synergizing the Transport across BBB

Liposomes can be functionalized with one or more ligands for improving the delivery of the encapsulated drug or plasmid to specific cells. Conjugating to multiple ligands can help perform multiple functions, e.g., one vector can facilitate brain tissue targeting, and another ligand can induce cellular uptake and/or intracellular translocation to specific cell compartments, like the nucleus for delivery of pDNA [146].

Conventional liposomes, composed of cholesterol and phospholipids, suffer from high plasma clearance and low transport across BBB. These liposomes can be surface modified with different ligands, like proteins, peptides and antibodies, for targeting specific receptors [147]. Xiang *et al.* showed increased tumor transport of chlorotoxin-modified PEGylated liposomes loaded with doxorubicin and greater inhibition of the tumor growth as compared to the unmodified liposomes [148]. In another study, Ying *et al.* [149] evaluated dual-targeting liposomes surface functionalized with *p*-aminophenyl-α-d-mannopyranoside and transferrin for crossing the BBB. The dual-modified liposomes showed enhanced transport across *in vitro* BBB and significantly decreased the C6 glioma tumor volume in rat models [149]. A recent report showed the comparison of five different targeting ligands (transferrin, RI7217, COG133, angiopep-2 and CRM197) in improving the brain delivery of liposomes [102]. RI7217 is the antibody targeted to mouse transferrin receptors; COG133 is an apo-E mimetic peptide targeted to the low density lipoprotein receptors (LDLR) on the surface of BBB; angiopep-2 can bind to the LDLR-related protein on the brain endothelial cells; and CRM197 can bind to the diphtheria toxin receptor (DTR). The results indicated that only CRM197 was able to bind to the brain endothelial receptors *in vitro*, while RI7217 showed maximum uptake into brain *in vivo*.

## 7. Conclusions and Future Prospects

The multi-ligand nanocarriers bear the potential to serve as safe and efficient non-viral vectors for the transport of desired therapeutic molecules across the BBB.

Specifically, a combination of CPP and receptor-targeting protein (e.g., transferrin) can serve as a promising platform for the design of novel drug and gene delivery vectors. In this review, we have described various challenges involved in the successful transport of therapeutic agents to brain. Transferrin is the most common receptor targeted for delivery of small molecules or proteins across BBB [16]. A combination of the receptor targeting properties of this protein and the efficient cell penetration property of the CPPs has shown significant improvement in brain delivery as compared to only single ligand receptor-targeting agents [17]. This platform can be further manipulated to employ different targeting ligands, like receptor binding amino acid sequences of antibodies and proteins for delivery to desired cells. These specific receptor binding sequences, in combination with short chain CPP sequences, are anticipated to augment the targeting and cellular delivery of lipid-based nanocarriers across different cellular barriers [17]. This approach could therefore be used to target numerous receptors, such as insulin, neuronal nicotinic acetyl choline, vascular endothelial growth factor receptors, *etc.*, for the treatment of diseases, like diabetes, Alzheimer’s disease, schizophrenia and tumors.

## Figures and Tables

**Table 1:** Features of some naturally-occurring cell-penetrating peptides [14,71,89,90].

CPP	Amino Acid Sequence	Net Charge	Cell Lysis Activity
pAntp_43–68_ (Penetratin)	RQIKIWFQNRRMKWKK	+8	No
SynB1	RGGRLSYSRRFSTSTGR	+6	Yes
SBP	MGLGLHLLVAAALQGAWSPKKKRKV	+6	No
SynB3	RRLSYSRRRF	+6	-
Transportan	GWTLNSAGYLLGKINLKALAALAKKIL	+4	No
FBP	GALFLGWLGAAGSTMGAWSQPKKKRKV	+6	-
TAT_48–60_	GRKKRRQRRRPPQ	+8	No

CPP, cell-penetrating peptide.

**Table 2:** Examples of drug and gene delivery vectors for transport across BBB.

Nanoparticles for Brain Delivery	Properties	References
Bolaamphiphilic cationic vesicles	High serum stability, efficient cell uptake and improved brain targeting	[117,118]
Poly(lactide-co-glycolide) (PLGA) nanoparticles	Biocompatible, biodegradable, efficient cellular uptake and delivery of therapeutic agents into cells	[119,120]
Angiopep-conjugated nanoparticles	Internalization by brain capillary endothelial cells, efficient cell uptake, transport across BBB and gene expression	[121,122]
CPP-modified Tf-liposomes	Biocompatible, efficient cell uptake, transfection, transport across BBB *in vitro* and *in vivo*	[16,17,18]
RVG peptide-conjugated nanocarriers	High serum stability, biocompatibility, efficient transfection *in vitro* and *in vivo*	[123,124]
Solid lipid nanoparticles	Biocompatible, efficient cell uptake and drug delivery *in vitro*, efficient brain delivery *in vivo*	[125,126,127]
TAT-liposomes	Efficient cell uptake, low cytotoxicity, improved brain targeting and penetration	[22,128]
Surfactant-coated nanoparticles	Efficient brain penetration and improved therapeutic efficacy	[129,130,131]
Antibody-conjugated nanoparticles	Significantly enhanced brain delivery, biocompatible, improved therapeutic efficacy	[132,133,134]
